# Can Untrained Patients Perform Their Own Skin and Soft Tissue Ultrasound Examination by Teleguidance?

**DOI:** 10.24908/pocus.v8i2.16454

**Published:** 2023-11-27

**Authors:** Ammar Saati, Arthur Au, Aditi U Joshi, Rebecca Davis, Frances Mae West, Resa E Lewiss

**Affiliations:** 1 Department of Cardiovascular Medicine Section of Vascular Medicine, Heart, Vascular and Thoracic Institute, Cleveland Clinic Foundation Cleveland, OH USA; 2 Department of Emergency Medicine, Thomas Jefferson University Philadelphia, PA USA; 3 Digital Health Intelligence, MDisrupt, Founder, Nagamed LLC; 4 Department of Internal Medicine, Thomas Jefferson University Philadelphia, PA USA; 5 Department of Internal Medicine, Division of Pulmonary, Allergy, & Critical Care Medicine, Thomas Jefferson University Philadelphia, PA USA; 6 Department of Emergency Medicine, University of Alabama at Birmingham Birmingham, AL USA

**Keywords:** handheld ultrasound, POCUS, tele-POCUS, SARS-CoV-2

## Abstract

**Objectives: **This pilot study aims to determine if patients untrained in performing ultrasound can self-scan to obtain images under remote clinician teleguidance during a simulated telehealth encounter. This study also seeks to describe the patients’ comfort level and barriers to performing an ultrasound examination on themselves using a handheld ultrasound device. **Methods: **This was a single center prospective observational cohort study conducted over a 4-month period in 2021. Patients were eligible if they had no prior training in the use of ultrasound and in the use of teleguidance. They voluntarily consented to participate at a single ambulatory internal medicine clinic. **Results: **20 participants were enrolled and underwent teleguidance to ultrasound their own skin and soft tissues at the antecubital fossae. Six second video clips were evaluated by 2 subject matter experts using the Point of Care Ultrasound Image Quality scale. A score >7 was considered adequate for diagnostic interpretation. The average score was 10.15/14, with a minimum score of 5/14, and maximum score of 14/14 and a standard deviation (SD) of 2.39 using a two tailed Z-score. Setting alpha at 0.05 the 95% CI was (5.47-14.83). **Conclusion: **In a pilot study of 20 participants with no ultrasound experience, untrained healthy volunteers were able to perform technically acceptable and interpretable ultrasound scans using teleguidance by a trained clinician.

## Introduction

The SARS-CoV-2 pandemic accelerated the use of telehealth with consumer adoption increasing from 11% in 2019 to 46% in 2020 1, 2. A telehealth visit often replaced an in-person office visit for infection control and safety to the patient and healthcare team. Telehealth, the use of technology for remote medical encounters, can be an efficient way to connect doctor and patient synchronously or asynchronously. “Store and forward,” a form of asynchronous telemedicine utilizes uploaded pictures by patients for evaluation by a clinician. This aids the patient evaluation and improves the diagnostic capacity of a virtual examination 3. A systematic review of meta-analyses from 2010 to 2019 demonstrated that telehealth can be equivalent or more clinically effective when compared to routine care 4. One study showed that a caregiver can assist with the telehealth encounter when technology, education, or aptitude is a concern and the patient cannot manage the technology and imaging functioning themself. Similarly, family member engagement in the telehealth encounter helps with a physical examination under clinician guidance 5.

Over the past 20 years, point of care ultrasound (POCUS) has proven to be an error-reducing tool, improving diagnostic accuracy for a variety of conditions 6, 7, 8. Large, heavy, and difficult to operate ultrasound systems have been replaced by smaller, portable, and more affordable POCUS devices, which connect to a smartphone or a tablet. Advances in this portable technology make ultrasound more available to clinicians and patients in a variety of practice environments. As technology continues to improve, these hand-held ultrasound devices (HUS) will be ubiquitous and affordable to clinicians and patients. Telehealth physicians are primed to begin incorporating POCUS imaging into patient encounters to expand diagnostic capabilities. Studies of patients infected with SARS-CoV-2 suggest that trained patients can scan their own lungs 9. Some HUS devices have a teleguidance feature, which allows real-time clinician guidance over video to assist the patient in image acquisition 10, 11. Artificial intelligence (AI) has also been developed for some HUS devices to assist novice sonographers in image acquisition 12. To date, it has not been determined how HUS can effectively be integrated to support the evaluation and diagnostic accuracy during a telehealth visit.

Sargsyan et al. studied the focused assessment with sonography for trauma (FAST) examination performed by astronauts with remote guidance with excellent clinical results 13. Jensen et al. evaluated the practical feasibility, performance, and acceptability of real-time supervision of tele-ultrasound. The authors found that distant supervision was feasible for both junior physicians and supervisors when applied to lung and cardiac ultrasound 14. These studies support that POCUS can be incorporated into a telemedicine program under the real-time guidance from POCUS experts 15. One case report described a patient infected with SARS-CoV-2. His clinicians monitored him from home based upon self-performed lung ultrasound examinations using a HUS device 16.

With the increased utility of telehealth and patient engagement with the use of HUS, we hypothesized that a POCUS trained clinician can remotely guide a patient to acquire clinically useful ultrasound images by self-scanning during a telehealth encounter. 

Skin and soft tissue infections including cellulitis and abscesses account for nearly 4.2 million emergency department visits annually 17. Clinicians evaluate soft tissue infections with visual inspection of the affected area for erythema, warmth, swelling and edema, followed by palpation of the area for warmth and fluctuance, suggesting an abscess or phlegmon. While the former may be indicative of cellulitis and be treated conservatively with antibiotics, abscesses may require an incision and drainage procedure for adequate source control. Studies show that POCUS aids in the accurate differentiation of cellulitis and abscess 18. The POCUS examination is typically straightforward where an affected part of the body is evaluated in two planes. An examination is then performed for comparison on the opposite side. The clinician looks for a cobble stoning pattern in the subcutaneous tissue concerning for cellulitis and a heterogeneous irregular bordered collection of fluid pattern suggestive of an abscess. Therefore, a single POCUS application, skin and soft tissue scan (STSS), was selected for this pilot study because cellulitis and abscess formation are common concerns prompting a visit for evaluation and because the examination tends to be straightforward.

## Materials and Methods

This was a prospective observational cohort study conducted from March to June 2021 at the Jefferson Internal Medicine Associates (JIMA) clinic in Philadelphia, PA. JIMA is an academic Internal Medicine primary care clinic with approximately 35,000 patient visits annually. The study was approved by the institutional review board committee.

### Selection of participants

Participants were drawn from patients with scheduled primary care visits at JIMA clinic. The principal investigator (PI = AS) attended a meeting with the primary care faculty and introduced the study with a request for assistance with patient enrollment. Each week, the JIMA physicians were contacted by the primary investigator (AS) via email. The email served as a reminder for the clinicians that the study was open for enrollment. At the end of the visit the JIMA doctor pre-screened healthy patient volunteers for enrollment. Patient inclusion criteria were: English speaking, age greater than 18, willing to participate and able to consent. The following patients were not eligible for inclusion: unstable vital signs, a rash over the area of interest, prisoners, and patients with HUS, POCUS, or teleguidance experience. The primary care physician, at the end of their regularly scheduled patient visit, would introduce the study opportunity. Interested participants were escorted to a simulated telehealth space within the clinic for consent and enrollment. The PI then confirmed that the patient met inclusion criteria and obtained written consent. Demographic variables were not collected.

### POCUS application

Five subject matter experts met and discussed a POCUS application felt to be straightforward for teleguidance. This application would serve as the example for the study. After three discussion sessions, the SSTS application was selected by consensus. The group decided that the medial forearm in the antecubital fossa area would serve as the anatomic region for patients to self-scan. 

### HUS Equipment

A Philips Lumify L12-4 Broadband linear array transducer (Koninklijke Philips N.V.) and a Samsung Galaxy Android tablet S6 (Samsung Electronics Co., Ltd.) constituted the HUS device. The Lumify SSTS application was pre-set, with gain and depth adjusted. For teleguidance, we used the REACTS (Remote Education, Augmented Communication, Training and Supervision) software (Innovative Imaging Technologies). This real-time, secure audio/video software enables a clinician to remotely guide another user performing a POCUS examination. 

### Scanning protocol

The authors developed a standardized script when communicating with the participants. In the telehealth simulated room, the PI handed the HUS device to the participant and activated the REACTS application. Then the PI left the patient and moved to a separate location in the JIMA clinic. The PI started the session by making sure that the audio and visual were clear and by introducing the participants to the ultrasound probe. The PI guided the participant on how to apply gel to the probe, identify the probe marker and how to apply the probe to their own body. The participant was then, visually instructed to apply the transducer at the antecubital fossa and to slide the probe proximal and distal in the transverse position. Once that step was successful the participant was instructed to annotate (label) the ultrasound images (right or left, transverse or longitudinal). After the annotation, the participant was directed to press the record icon on the Samsung tablet to save a 10 second video clip. Participants performed self scan imaging of the right and left antecubital fossa in transverse and longitudinal planes. Participants were not taught or expected to adjust depth or gain (Video S1 and Video S2). The images were saved in a de-identified manner to the Samsung tablet and subsequently uploaded to a secured shared server at Thomas Jefferson University Hospitals. 

### Outcome measures

The quality of the archived images was evaluated by two POCUS physician experts. They were not involved in participant enrollment and were blinded to each other's scores. They used a scoring system adapted from Dessie A, et al. to quantify the quality of the images 19. The scoring system consists of 3 categories: 1) technical (probe choice, depth, gain), 2: scanning skills (probe control, anatomy/ landmarks), and 3) interpretability (labeling and completeness). For each subcategory the expert assigned a score of Poor (0 point), Adequate (1 Point), or Ideal (2 point). Fourteen (14) was the highest score possible. A score of <7 was considered an inadequate study. The start time began the moment the participant established teleguidance with the PI. The end time was the moment the PI discontinued the teleguidance. Individual scores were collected through RedCap and calculated automatically. Participants were asked for feedback, and this was noted by the enrolling clinician. A descriptive statistical analysis was generated, and a two tailed Z score was calculated to measure the confidence interval (CI) of the average score. Finally, a 1 tailed t-test was calculated to compare the average score between the two evaluators.

## Results

Twenty-two participants were referred by the JIMA physicians. Two participants did not meet inclusion criteria and were excluded due to prior POCUS experience. Twenty final participants performed teleguided self-scanning. The two POCUS credentialed evaluators (AA, FMW) reviewed all images for the 20 participants. 

The L12-4 Broadband linear array transducer was used by all the participants and received an ideal score representing 100%. With regard to depth the majority 62.5% had an ideal depth. The preset for gain was already set to soft tissue study, 20% had adequate gain for visualization of the area of interest and 80% had an ideal gain setting. Probe control was excellent in 30%, fair in 35% and poor in 35% of studies evaluated. Anatomy landmark recognition was excellent in 35%, fair in 52.5%, and poor in 12.5% of studies. Participant labeling was ideal in 45%, adequate in 42.5%, and poor in 12.5% of clips. For the completeness of viewing all clips 47.5% were ideal, 37.5% were adequate, and 15% were poor (Table 1).

**Table 1:** Graded image quality of point of care ultrasound examinations obtained via teleguidance. Two POCUS expert clinicians scored the images for N = 20 participants. The quality scoring system falls into three categories: Technical, Scanning skills, and Interpretability. Permission granted from Almaz Dessie MD (6/3/2021) [19].

**Technical**			
-	Inadequate	Adequate	Ideal
1- Probe choice	0	0	100%
2- Depth	10%	27.5%	65.5%
3- Gain/preset	0	20%	80%
**Scanning Skills**			
-	Poor	Fair	Excellent
1- Probe control	35%	35%	30%
2- Anatomy Landmarks	12.5%	52.5%	35%
**Interpretability**			
-	Inadequate	Adequate	Ideal
1- Labeling	12.5%	42.5%	45%
2- Completeness	15%	37.5%	47.5%

**Figure 1:**
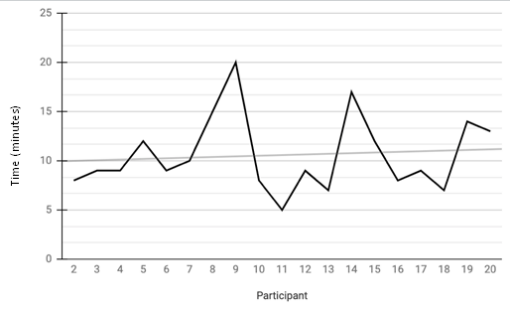
Line graph demonstrating the amount of time for each participant teleguidance encounter. In this figure, time in minutes is plotted along the Y axis. The participant is plotted on the X axis (n = 20). Participant 1 is the first patient enrolled. Participant 20 is the last patient enrolled. The encounters took an average of 10.6 minutes. The shortest encounter took 5 minutes. The longest encounter took 20 minutes.

The average duration spent scanning was 10.6 minutes with a minimum of 5 minutes and a maximum of 20 minutes (Figure 1).

The average total score was 10.15/14, minimum score 5/14, and maximum score 14/14 with a standard deviation (SD) of 2.39. Using a two tailed Z-score, setting alpha at 0.05 the 95% CI was (5.47-14.83). 

**Figure 2:**
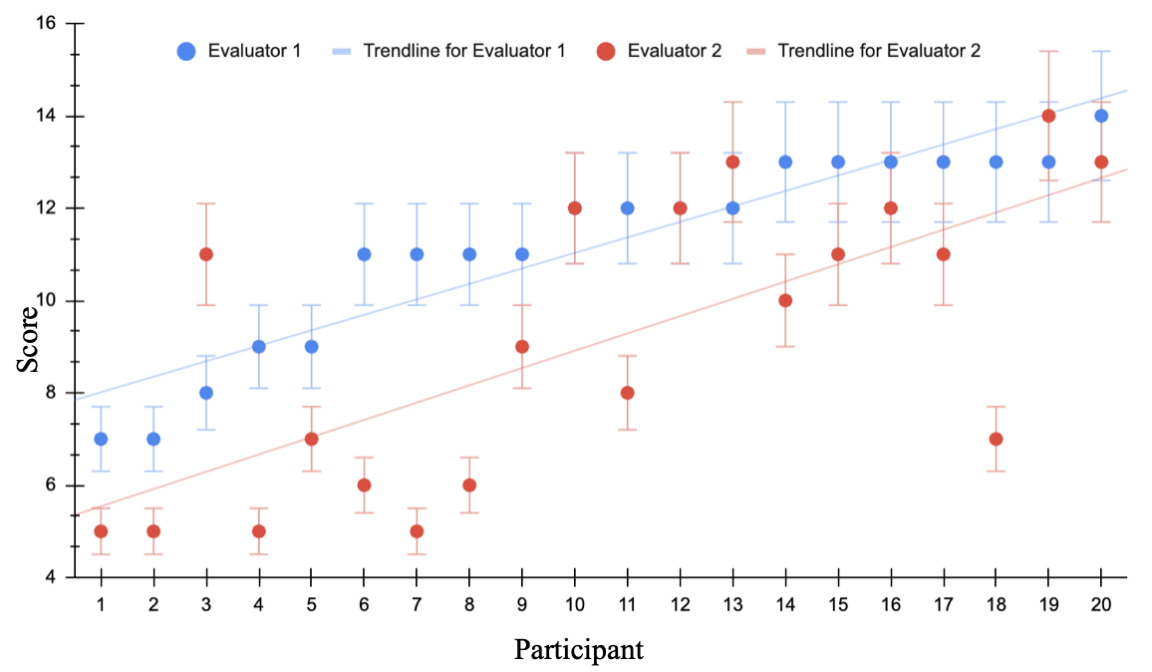
Scatter plot of each participant with their corresponding score for image quality of POCUS obtained via teleguidance. A maximum of 14 possible points could be obtained. Blue represents Evaluator 1. Red represents Evaluator 2. The X axis illustrates a participant with random assignment from 1 to 20. The Y axis represents the total score assigned by the evaluator.

For evaluator 1 the average score was 11.2/14 with a SD 2.11 and a 95% CI was (7.04-15.35). Evaluator 2 had an average score of 9.1/14; SD 3.14 and a 95% CI was (2.93-15.26). The results indicated a statistically significant difference between the mean scores of evaluator 1 and evaluator 2, as determined by a one-tailed T-test with a P value of 0.0005 (Figure 2). 

Participants' provided feedback predominantly about the challenge of labeling the images. One participant felt the gel was difficult to apply. One participant commented that the application would be easier for a younger generation. Another participant forgot their eyeglasses. And finally, one patient was hard of hearing and found teleguidance difficult. Anecdotally, participants expressed excitement about the technology. They expressed optimism that POCUS examinations performed with the assistance of teleguidance had promise to augment patient care.

## Discussion

The primary aim of this study was to determine whether participants with no POCUS or teleguidance experience could perform an adequate and interpretable STSS of the antecubital fossae. We found that 85% of participants were able to obtain adequate images using the POCUS Image Quality Assessment Tool 19. 

The SARS-CoV-2 pandemic accelerated the use, acceptance, and necessity for telehealth 20. The use of POCUS has been utilized by emergency physicians for three decades 21, allowing minimally invasive, quickly gathered information to improve diagnostic accuracy. The use of ultrasound by patients could enhance telemedicine care by improving evaluation and diagnostics, with studies showing feasibility with teleguidance. 

It was previously observed that participants younger than 80 years old who use the internet daily can obtain ultrasound images with minimal instruction 22. In our study, one participant noted that the application might be easier for a younger generation with more exposure to tablets and their associated application software. The average scan time duration of 10 minutes was thought to be reasonable and was acceptable to the participants. 

Overall, these results suggest that HUS can be used by untrained participants over a telehealth encounter with instruction. Teleguidance has the potential to enhance remote care and improved access for patients in rural or resource limited and disaster areas. Teleguidance can also be utilized during telemedicine encounters to assist an untrained clinician to obtain ultrasound images for consultation and education. Further studies are needed to evaluate if patient-performed HUS studies are attainable for diagnostic utility. Such findings might suggest an expansion of ultrasound training and instruction in tele guidance. 

In conclusion, participants in this pilot study were able to obtain adequate SSTS, utilizing HUS with teleguidance by a POCUS trained physician. This practice could prove valuable in telemedicine evaluations and diagnoses. 

## Limitations

There are several limitations to this pilot investigation. This was a single center study and the participant enrollment was small in number and performed by one clinician. Participants were not randomized. Since skin and soft tissue ultrasound was the one application tested, the patient self-scan may not be generalizable to other ultrasound applications. It is unclear why the evaluator scores differed. Future studies could include more evaluators or more participants to see if this is a true discrepancy. In addition, more time could have been dedicated to educating the evaluators. Participants were observed to scan quickly over the anatomic area of interest despite instructions to move the probe slowly. All studies were of normal soft tissue. It is unclear how well patients would be able to self-scan over an affected area of cellulitis or abscess.

 Although the average time to perform the POCUS examination was 10 minutes, it is not clear how this would affect the workflow of a telemedicine encounter. This study also made use of a simulated telemedicine encounter without the barriers of connectivity, devices, or ability to upload images. If telemedicine encounters and HUS are used at home, further studies on home encounters are needed. Future investigations are needed with a larger sample size and more evaluators. 

## Disclosures

This work has not been presented at meetings, no grant support was received. REL serves on the Medical Advisory Board for EchoNous, on the board of PURE, on the board of Society for Clinical Ultrasound Fellowships (SCUF), and previously received equipment support from Phillips Healthcare and Butterfly Network. Otherwise, there are no disclosures of relevant commercial interests. 

## Supplementary Material 

 Video S1 Self scan of a patient’s right forearm in the longitudinal view with a fast scan sweep. The probe loses contact with the skin at the beginning and end of the clip.

Video S2 Self scan of a patient’s right forearm in the transverse view with a sweep through the tissues. There is delayed contact with the skin resulting in an abbreviated clip.

## References

[R214823129557748] Bestsennyy O, Gilbert G, Harris A, Rost J Telehealth: A quarter-trillion-dollar post-COVID-19 reality?. https://www.mckinsey.com/industries/healthcare-systems-and-services/our-insights/telehealth-a-quarter-trillion-dollar-post-covid-19-reality?cid=eml-web.

[R214823129557755] Bashshur R, Doarn C R, Frenk J M, Kvedar J C, Woolliscroft J O (2020). Telemedicine and the COVID-19 Pandemic, Lessons for the Future. Telemed J E Health.

[R214823129557740] Schwamm L H (2014). Telehealth: seven strategies to successfully implement disruptive technology and transform health care. Health Aff (Millwood).

[R214823129557752] Snoswell C L, Chelberg G, Guzman De, Haydonhh K R, Thomas E E, Caffery L J, Smith A C (2021). The clinical effectiveness of telehealth: A systematic review of meta-analyses from 2010 to 2019. J Telemed Telecare.

[R214823129557747] Benziger C P, Huffman M D, Sweis R N, Stone N J (2021). The Telehealth Ten: A Guide for a Patient-Assisted Virtual Physical Examination. Am J Med.

[R214823129557750] Gaspari R, Weekes A, Adhikari S, Noble V E, Nomura J T, Theodoro D, Woo M, Atkinson P, Blehar D, Brown S M, Caffery T, Douglass E, Fraser J, Haines C, Lam S, Lanspa M, Lewis M, Liebmann O, Limkakeng A, Lopez F, Platz E, Mendoza M, Minnigan H, Moore C, Novik J, Rang L, Scruggs W, Raio C (2016). Emergency department point-of-care ultrasound in out-of-hospital and in-ED cardiac arrest. Resuscitation.

[R214823129557753] Hilsden R, Leeper R, Koichopolos J, Vandelinde J D, Parry N, Thompson D, Myslik F (2018). Point-of-care biliary ultrasound in the emergency department (BUSED): implications for surgical referral and emergency department wait times. Trauma Surg Acute Care Open. Jul.

[R214823129557743] Kim S G, Jo I J, Kim T, Hwang S Y, Park J H, Shin T G, Sim M S, Cha W C, Yoon H (2019). Usefulness of Protocolized Point-of-Care Ultrasonography for Patients with Acute Renal Colic Who Visited Emergency Department: A Randomized Controlled Study. Medicina (Kaunas).

[R214823129557757] Kirkpatrick A W, Mckee J L, Moeini S, Conly J M, Ma I W Y, Baylis B, Hawkins W (2021). Pioneering Remotely Piloted Aerial Systems (Drone) Delivery of a Remotely Telementored Ultrasound Capability for Self Diagnosis and Assessment of Vulnerable Populations-the Sky Is the Limit. J Digit Imaging.

[R214823129557745] Philips Healthcare Tele-ultrasound solution: Lumify with Reacts. Philips Healthcare.

[R214823129557749] Butterfly Network Teleguidance. Butterfly Network.

[R214823129557741] Kosmos Point-of-Care Ultrasound AI-Powered, Ultraportable Kosmos. Kosmos Point-of-Care Ultrasound.

[R214823129557744] Sargsyan A E, Hamilton D R, Jones J A, Melton S, Whitson P A, Kirkpatrick A W, Martin D, Dulchavsky S A (2005). FAST at MACH 20: clinical ultrasound aboard the International Space Station. J Trauma.

[R214823129557754] Jensen S H, Duvald I, Aagaard R, Primdahl S C, Petersen P, Kirkegaard H, Weile J (2019). Remote Real-Time Ultrasound Supervision via Commercially Available and Low-Cost Tele-Ultrasound: A Mixed Methods Study of the Practical Feasibility and Users' Acceptability in an Emergency Department. J Digit Imaging.

[R214823129557758] Britton N, Miller M A, Safadi S, Siegel A, Levine A R, Mccurdy M T (2019). Tele-Ultrasound in Resource-Limited Settings: A Systematic Review. Front Public Health.

[R214823129557742] Pivetta E, Girard E, Locascio F, Lupia E, Martin J D, Stone M (2019). Self-Performed Lung Ultrasound for Home Monitoring of a Patient Positive for Coronavirus Disease. Chest.

[R214823129557759] Prusakowski M K, Kuehl D R (2015). Trends in emergency department management of skin abscesses. Am J Infect Control.

[R214823129557739] Subramaniam S, Bober J, Chao J, Zehtabchi S (2016). Point-of-care Ultrasound for Diagnosis of Abscess in Skin and Soft Tissue Infections. Acad Emerg Med.

[R214823129557756] Dessie A S, Calhoun A W, Kanjanauptom P, Gilbert G E, Ekpenyong A, Lewiss R E, Rabiner J E, Tsze D S, Kessler D O (2022). Development and Validation of a Point-of-Care-Ultrasound Image Quality Assessment Tool: The POCUS IQ Scale. J Ultrasound Med.

[R214823129557746] Bashshur R, Doarn C R, Frenk J M, Kvedar J C, Woolliscroft J O (2020). Telemedicine and the COVID-19 Pandemic, Lessons for the Future. Telemed J E Health.

[R214823129557760] (2016). Emergency Ultrasound Imaging Criteria Compendium.. Ann Emerg Med.

[R214823129557751] Resnikoff P M, Shi R, Bagsic S R Spierling, Kimura B J (2021). The Novel Concept of Patient Self-Imaging: Success in COVID-19 and Cardiopulmonary Disorders. Am J Med.

